# Barriers and needs in mental healthcare of adults with autism spectrum disorder in Germany: a qualitative study in autistic adults, relatives, and healthcare providers

**DOI:** 10.1186/s12888-023-05026-x

**Published:** 2023-07-21

**Authors:** Sophia Dückert, Petia Gewohn, Hannah König, Daniel Schöttle, Alexander Konnopka, Pascal Rahlff, Frank- Erik, Kai Vogeley, Holger Schulz, Nicole David, Judith Peth

**Affiliations:** 1 Present Address: Department of Medical Psychology, University Medical Center Hamburg-Eppendorf, Martinistraße 52, 20246 Hamburg, Germany; 2 Department of Psychiatry and Psychotherapy, University Medical Center Hamburg- Eppendorf, Hamburg, Germany; 3 Department of Health Economics and Health Services Research, University Medical Center Hamburg-Eppendorf, Hamburg, Germany; 4 Department of Psychiatry, Faculty of Medicine and University Hospital Cologne, University of Cologne, Cologne, Germany

**Keywords:** Autism spectrum disorder (ASD), Adults, Mental healthcare, Qualitative research, Participatory research

## Abstract

**Background:**

Autism refers to a neurodevelopmental condition with characteristic impairments in social interaction and communication, restrictive and repetitive behaviors, as well as difficulties in sensory information processing and daily living skills. Even though symptoms persist from early childhood throughout the lifespan and often require long-term support, there is a lack of mental health services that sufficiently meet the needs of autistic adults. Previous evidence suggested individual, professional and structural barriers to healthcare for autistic adults. Here, using a peer research approach, we sought to systematically investigate barriers and needs in mental healthcare of autistic adults in Germany at the three relevant levels (individual, professional, structural) and from three relevant perspectives (autistic adults, relatives and healthcare providers), in order to obtain specific recommendations for optimized healthcare.

**Methods:**

Maximum variation sampling was used to account for the complexity of the research field. Semi-structured, open-ended interviews were conducted with autistic adults (*n* = 15) and focus groups with relatives/partners (*n* = 12), and healthcare providers of several professions (*n* = 15). Data analysis was performed using the codebook approach of thematic analysis.

**Results:**

Poor mental healthcare of autistic adults in Germany was characterized by six central and overarching themes: (i) lack of knowledge about autism, (ii) a need for increased participation/involvement, (iii) consideration of autism-specific needs in treatment, (iv) lack of services, (v) limited access to services, and (vi) improvement of stakeholder collaboration. Themes were similarly reported across participants, emphasizing dissatisfaction in all stakeholders.

**Conclusions:**

We identified major barriers to mental healthcare for autistic adults in Germany that affect autistic adults, but are also of concern to relatives and healthcare providers. Our results point to specific and generic areas for improvement, independent of stakeholder perspectives, which could guide future development of needs- and evidence-based services, recommendations and guidelines of mental healthcare for people with autism across the lifespan.

**Trial registration:**

This study protocol was preregistered at the Open Science Framework (https://osf.io/5x8pg).

**Supplementary Information:**

The online version contains supplementary material available at 10.1186/s12888-023-05026-x.

## Background

Autism spectrum disorder (ASD) is a pervasive neurodevelopmental disorder with a global prevalence of approximately 1% [1]. Manifestations of autism include characteristic impairments in social interaction and communication, as well as restrictive and repetitive behaviors, including abnormalities in sensory information processing, and difficulties in critical domains of daily living [2]. Previous evidence showed increased rates of comorbid somatic diseases, such as epilepsy, diabetes, gastrointestinal, cardiovascular and respiratory diseases [3–5], and psychiatric conditions [4, 5]. Psychiatric comorbidities affect 75–80% of autistic adults^1^, with the most common including anxiety and mood disorders, attention-deficit/hyperactivity disorder (ADHD), and schizophrenia [3, 5]. Furthermore, increased rates of premature mortality [4] and risk of committing suicide and dying by suicide [6, 7] have been reported. As such, autistic adults often require a broad variety of medical as well as mental healthcare services, including social services like supported employment [8]. As autism persists across the lifespan, the need for support also persists across the transition from childhood to adult services. Nevertheless, the majority of specialized autism services have focused on autistic children [9] or autistic adults with an accompanying intellectual disability (ID; IQ < 70), with little attention paid to the needs of autistic adults without ID. Nevertheless, half to two-thirds of autistic adults do not have an ID [10, 11], with prevalence rates increasing over the last few years [12]. However, affected adults often report multiple health conditions, severe limitations in practical living skills and a substantial need for support [13], while –at the same time—having difficulties to accessing or finding appropriate services [5, 14] compared to the general population [15]. As a result, autistic adults reported higher odds of unmet healthcare needs, showed a lower general healthcare and ineffective utilization of services [16, 17]. These, in turn, feed into a vicious circle associated with declines in psychosocial functioning or health-related quality of life and higher rates of comorbidity, chronicity, and suicidality [5, 18–20]. Furthermore, the unmet healthcare needs of autistic adults lead to increased economic costs for the healthcare system, autistic adults and their families [8]. In addition to the financial burden, families and partners of autistic adults typically compensate for the lack of support, often facing high levels of responsibility and stress as reflected in, for example, increased time and emotional burden [21, 22], anxiety and depression [23]. Thus, relatives and partners of autistic adults also face healthcare needs that are currently massively underserved [24, 25].

This evidence emphasizes the need to develop more effective healthcare structures that make best use of scarce societal resources and to investigate the mechanisms underlying ineffective healthcare of autistics adults. Internationally, some barriers have already been suggested, which hinder adequate and effective healthcare for autistic adults. For the U.S., for example, Nicolaidis and colleagues [26] suggested barriers to general healthcare for autistic patients at three different levels: the individual level, the professional level, and the structural or systemic level (for purpose of simplification, mentioned as “structural level” below). Contributing factors at an individual level include impairments due to autism-related difficulties, e.g. hypersensitivity in physical examinations [27, 28] or difficulties in verbalizing medical conditions or pain [29]. The professional level, or provider level, refers to, for example, healthcare providers’ (lack of) knowledge about autism [26] or providers’ degree of flexibility in contact with autistic patients [30], and structural level factors generally refer to the lack of availability or accessibility of healthcare services [28, 29]. Similar barriers have been suggested for the United Kingdom [15] or Canada [29]. However, evidence on mental healthcare of autistic adults is limited, especially in Germany. The German healthcare is defined as “social insurance system” [31] and is based on the welfare state, where a statutory or private health insurance can be opted for. Both insurance systems are separated along different organizational, regulative, and financial sectors. Due to separate legislation within public health services (i.e. inpatient, outpatient, and long-term care), there is a fragmentation of service provision [32], which could, for example, lead to healthcare services not being funded. According to data of the World Health Organization (WHO), Germany’s health expenditure in 2018 ranked second among European countries (11.4% of Gross Domestic Product (GDP)) [32]. Despite this resource and an established system of supply, the German Guidelines Report “Autism spectrum disorders in childhood, adolescence and adulthood Part 2: Therapy“ critically remarked the lack of epidemiological or healthcare research in Germany, and the importance of analyzing care pathways, barriers and accessibility of facilities in order to make suggestions for improvement [33]. This is in line with the recently published Lancet Commission on the future of care and clinical research in autism, which emphasized the need for developing and implementing a high-quality, evidence-based, and cost-effective healthcare model [34, 35], tailored to meet the individual needs of this heterogeneous population [1, 26].

Within the realms of the bigger research project *BarrierfreeASD* [36], the present study aimed at assessing the current state of mental healthcare for autistic adults in Germany. Specifically, in a three-by-three fashion, we sought to systematically investigate barriers and needs in mental healthcare of autistic adults at the three relevant levels (individual, professional, structural) and from three relevant perspectives involving all relevant stakeholders into research (autistic adults, relatives/partners, healthcare provider) in order to gather a comprehensive picture. For this purpose, one-to-one interviews with autistic adults and focus groups with relatives/partners and healthcare providers from different professional groups were conducted. In line with recommendations for participatory research on autism [37, 38], the entire study was planned, conducted, and analyzed by a neurodivergent team involving autistic adults, as well as in cooperation with autism-, relative-, and service-related associations.

## Methods

This qualitative study was conducted as part of the project *BarrierfreeASD* [36]. Ethical approval was received by the Local Psychological Ethics Commission at the Center for Psychosocial Medicine of the University Medical Center Hamburg-Eppendorf (#LPEK-0227; Dec. 2020) and the study was carried out in accordance with the Declaration of Helsinki. All participants provided consent prior to participating. Participants did not receive compensation for expenses. The *BarrierfreeASD* project was preregistered at Open Science Framework (https://osf.io/5x8pg).

### Participants

Three groups of stakeholders were interviewed: (i) adults (18 + years) with a diagnosis of childhood autism, Asperger syndrome, atypical autism, or autism spectrum disorder, without ID (IQ > 70), (ii) first or second-degree relatives or partners (18 + years) of autistic adults (not necessarily related to the autistic adult sample), (iii) healthcare providers from varying professional backgrounds and healthcare sectors (see below). A total sample size of *N* = 45 (each stakeholder group *n* = 15) was aimed for in order to adequately address the research questions [39].

In order to account for the heterogeneity and complexity of the research field, maximum variation sampling was conducted, a purposive sampling strategy that obtains fundamental understanding through diverse groups of individuals and perspectives [40, 41]. Furthermore, exponential discriminative snowball sampling was applied to access susceptible populations [42]. Within the group of autistic adults, a purposive sample was drawn to achieve the broadest possible diversity in terms of age at diagnosis, living situation, and support needs. The same criteria were applied in the group of relatives of autistic adults. In the sample of healthcare providers, the maximum variation was achieved in terms of their experience in the treatment of adult autistic persons and their professional background (e.g. primary care, pediatrics, adult and child psychiatry or psychotherapy, occupational therapy, speech therapy). Nonspecific recruitment criteria for all three groups were age, sex, and place of residence, respectively place of work within the group of healthcare providers.

Participants were recruited throughout Germany via the study’s collaborating network of cooperating partners, publicly available contacts from autism-related associations (including self-help and caregiver groups), healthcare associations (medical chambers, therapist associations, etc.), outpatient clinics, as well as social media.

Autistic adults (*n* = 15) averaged 38.9 years of age (Range = 22–58; see Table 1) and 60% were male (*n* = 9). Participants reported to have Asperger’s syndrome (86.7%; *n* = 13) or atypical autism (13.3%; *n* = 2) and most of them received their formal diagnosis in adulthood (80%; *n* = 12). The average time until correct ASD diagnosis was 8.1 years (Range = 0.5–45). Every participant reported at least one psychiatric or neurological co-morbidity, while 40% (*n* = 6) reported two co-morbidities, 13.3% (*n* = 2) reported three, and one autistic adult reported five co-morbidities (6.7%). The most frequently reported psychiatric co-morbidities were depression (64.3%; *n* = 9), ADHD (42.9%; *n* = 6), and anxiety disorders (28.6%; *n* = 4).

**Table 1:** Demographic characteristics

	Autistic adults (*N* = 15)	Relatives/ partners (*N* = 12)	Healthcare providers (*N* = 15)
	*N* (%)/ *M* (*SD*)	*N* (%)/ *M (SD)*	*N* (%)/ *M (SD)*
Demographic characteristics			
Age at participation (years)	38.9 (12.5)	54.3 (9.5)	49.8 (10)
Gender (male)	9 (60)	2 (16.7)	2 (13.3)
Marital status			
single	11 (73.3)		
relationship	4 (26.7)	12 (100)	
Employment *^1^			
full-time	3 (20)	6 (50)	
part-time	4 (26.7)	3 (25)	
minor	1 (6.7)	1 (8.3)	
not employed	2 (13.3)	2 (16.7)	
Region of Germany *^2^			
North	7 (46.7)	7 (58.3)	6 (40)
East	1 (6.7)	0	5 (33.3)
South	5 (33.3)	2 (16.7)	1 (6.7)
West	2 (13.3)	2 (16.7)	3 (20)

*Note. N*: sample size; *M*: mean, *SD*: standard deviation; *^1^Missing data of five autistic adults; *^2^Missing data of one relative/partner; One healthcare provider specified two regions

In the group of relatives/ partners (*n* = 12) the mean age were 54.25 years (Range = 32–65), and most were female (83.3%; *n* = 10). The majority of the participants were parents or people under legal custody (75%; *n* = 9) and the others were partners (33.3%; *n* = 4). In the group of healthcare provider, the mean age was 49.8 years (Range = 33–71) and most of them were female (86.7%; *n* = 13). Healthcare providers reported different professions: nine were psychotherapists (60%), two were psychiatrists (13.3%), and one each were psychologist, pediatrician, occupational therapist, and art therapist (each 6.7%). The average experience of working with autistic individuals were 16.93 years (Range = 5–43). 80% reported working with autistic adults in their daily business (*n* = 12). The majority of the healthcare providers reported providing counselling (93.3%; *n* = 14), diagnostics (80%; *n* = 12), and/or psychotherapy (individual therapy = 73.3%; *n* = 11; group therapy = 60%; *n* = 9).

### Data collection

First, a systematic literature search of national and international evidence on mental healthcare in autistic adults was conducted to identify previously published barriers, facilitators, and needs in order to develop guideline questions. Because of the limited evidence on mental healthcare for autistic adults, we employed an exploratory qualitative approach with broad guideline questions to obtain a comprehensive and deep understanding of participants’ mental healthcare experiences [43]. Three interview guides were developed, tailored to each stakeholder group. All interview guides contained identical questions about barriers, facilitators, and needs in healthcare, especially with focus on mental healthcare, of autistic adults on an individual, professional, and structural level in general and while transitioning into adulthood, and recommendations for improving mental healthcare for autistic adults. The interview guide for autistic adults was developed in collaboration with peer researchers of the *BarrierfreeASD* study and included additional questions about their diagnosis and possible psychiatric or psychotherapeutic treatment. Relatives’ interview guide also included questions about the use of services especially for relatives (such as self-help groups for relatives). Healthcare providers were also asked about severity-based measures in the mental healthcare of autistic adults. Interview guides where piloted twice, once internally with study staff and once with an external psychologist.

In previous studies, participants with autism preferred one-to-one interviews [44]. Therefore, data collection in autistic adults (*n* = 15) was conducted via semi-structured open-ended interviews, whereas relatives/ partners of autistic adults (*n* = 12) and healthcare providers (*n* = 15) participated in semi-structured focus groups. Data collection was conducted by two trained researchers (SD & PG, psychologists with expertise in interviewing autistic adults). An external psychologist maintained an observational protocol during the focus groups.

Because of the Covid-19 pandemic, interviews and focus groups were performed online enabled by an online video communication provider. To accommodate individual specifics in communication, autistic participants were also given the opportunity to answer interview questions in writing (*n* = 3), via chat or phone (*n* = 3) or to include a supporting person. Interviews lasted approximately 45–60 min and focus groups about 100–120 min, both were audio-recorded and transcribed orthographically. Identifying features in the transcription were anonymized prior to data analysis.

### Data analysis

A thematic analysis was conducted, a six-phase approach to find repeated patterns of meaning across a set of data [45], following the common procedure: familiarization with data, generating initial codes, developing themes, reviewing themes, defining and naming themes, and producing the report. This approach was used independently for each data source (interviews or focus groups). MAXQDA 2020 [46] was used for data management and analysis. A mixed inductive-deductive codebook approach at a semantic level was applied. This approach is suitable for describing and summarizing qualitative data and participants’ views on a specific topic [47]. For this purpose, codes were generated both deductively (developed from the existing literature on the mental healthcare of autistic adults) and inductively (derived from the qualitative data). All codes were combined into a codebook and had clinical or policy implications for the mental healthcare of autistic adults. Finally, these codes were used to identify themes (i.e. main issues, complex concepts). Two authors as well as two student research assistants completed a training program for thematic analysis and performed the coding procedure. To verify interpretation of the data and ensure inter-rater reliability, codes were discussed with the multidisciplinary study team, and the themes were developed iteratively.

Using a participatory approach, two autistic peer workers as well as study’s collaboration network, including autism-related and family caregiver-related associations, were involved in developing the interview guides, the recruitment of participants, and the data analysis.

## Results

Data analysis for the three stakeholder groups revealed a variety of barriers, facilitators, and needs in the healthcare of autistic adults in Germany, focusing on medical and psychotherapeutic care. In addition, recommendations for improving healthcare were identified. This resulted into six group-overarching themes with several subthemes that are outlined below: (1) lack of knowledge about autism, (2) a need for increased participation/ involvement, (3) consideration of autism-specific needs in treatment, (4) lack of services, (5) limited access to services, (6) improvement of stakeholder collaboration (see Table 2). The majority of themes and subthemes were reported equally across the three stakeholder groups. Differences are reported within the description of the themes.

**Table 2:** Overview of themes and subthemes

Theme	Subtheme	Example quotes
Lack of knowledge about autism		
	Stigma about ASD in society	“[…] public and media often have a very distorted picture of what autism actually is, and especially the area of high-functioning is extremely difficult” (autistic adult; ibew6)
	Lack of knowledge among healthcare providers	“Well, most people have heard of autism […] but very few people have any idea about autism, even the doctors, even the psychiatrists.“ (autistic adult; ibew10)
	Research about ASD in adulthood and women	“[…] there are no good screening tools at all.” (healthcare provider; B2)
A need for increased participation/ involvement		“It is important trying to have a stronger exchange with family members” (autistic adult; ibew8)
Consideration of autism-specific needs in treatment		
	Difficulties in executive functions and self-management	“[…] estimating how long it will take me to get there, even with public transportation, because I don’t drive a car. […] Or, in general, not to take on too much for one day and to estimate how long I have to plan for this appointment.” (Autistic adult; ibew2)
	Need for consistency and transparency	“The biggest challenge is when I go to a new physician where I don’t know the environment, the practice, everything.” (autistic adult, ibew5)
	Autism-specific sensory sensitivities	“Noises are very unpleasant and hardly bearable […]” (autistic adult; ibew2)
	Difficulties in verbal and nonverbal communication	“[…] they like to be on the matter level and tend to communicate information and the emotional stuff doesn’t get carried along” (autistic adult; ibew3)
Lack of services		
	Transitioning	“What would have been great is having someone by your side through transition from adolescence to adulthood, who has been by their side for a long time” (relative; I3)
	Diagnostic services	“The waiting time for a diagnosis has grown over two years.” (autistic adult; ibew10)
	Psychotherapeutic services	“There are not enough diagnostic and therapeutic centers at all.” (healthcare provider; T5)
	Inpatient services	“In inpatient healthcare of people with autism spectrum disorders, […] there are very, very few offers” (healthcare provider; B2)
	Low-threshold services	“There is only one telephone emergency service for autistic people in Germany and that is a voluntary one.” (healthcare provider; T3)
	Assistance services	“In my opinion, even a high-functioning autistic person like me needs some kind of lifelong assistance service” (autistic adult; ibew 11).
Limited access of services		
	Lack of transparency about healthcare services	“Access to services is so massively divided among the various social systems by law that it is incredibly difficult for me to even find out who is responsible for me and from whom I have to apply for what.” (autistic adult; ibew4)
	Funding	“[…] an autism therapy is usually not funded by the health insurance, because with health insurance financing there is always this healing aspect involved” (autistic adult; ibew2)
Improvement of stakeholder collaboration		“The dream of an interdisciplinary team, a place where physicians and therapists work together in a practice, accompanied by people who are well informed and well connected and can give information, kind of an autism support center […]” (relative; I7)

*Note.* ASD: Autism Spectrum Disorder

**Fig. 1:**
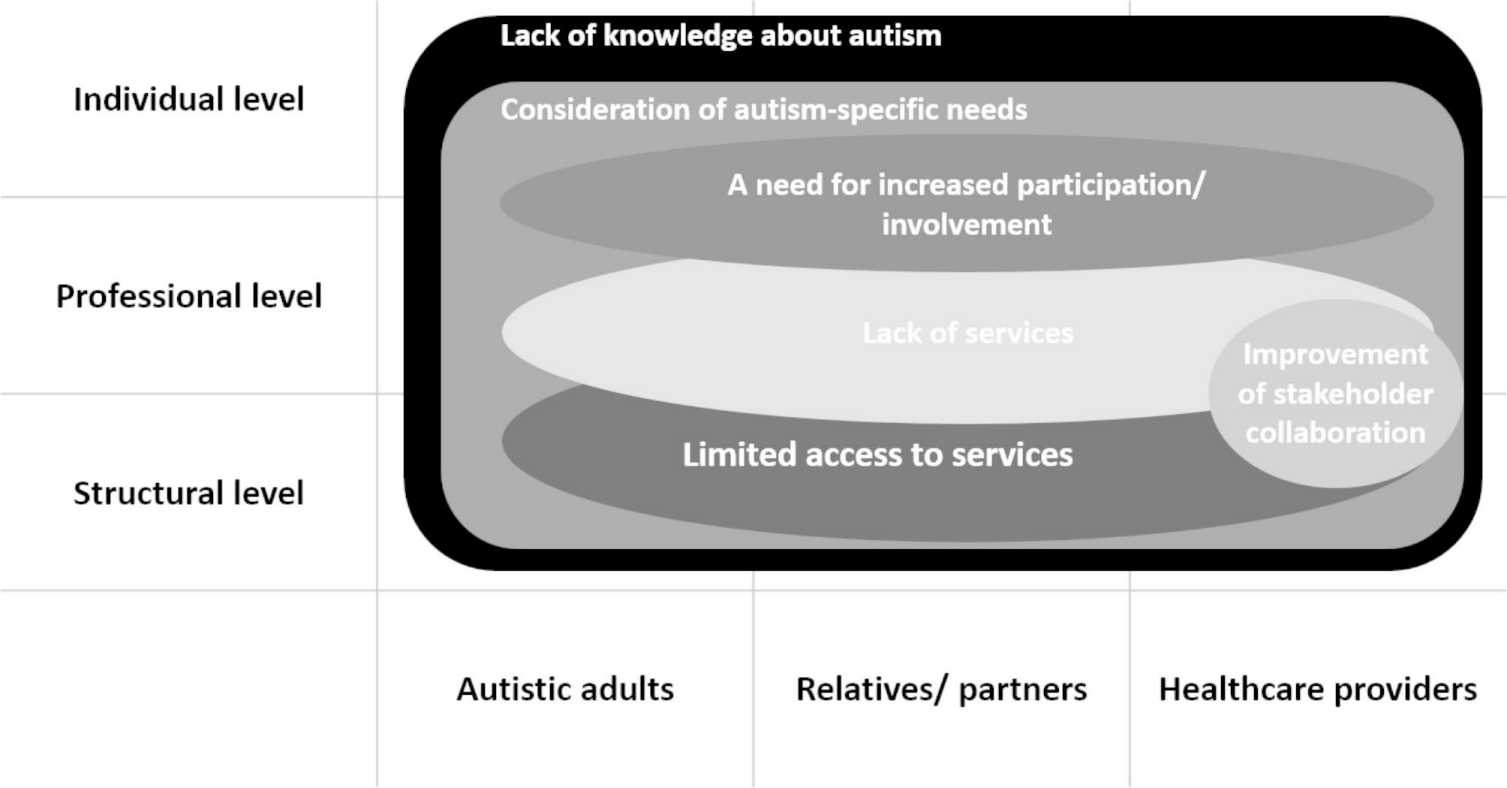
Simplified visualization of themes related to level and group of stakeholders

Although interviews and focus groups were structured to assess barriers and needs per level (individual, professional, structural) across groups, the resulting themes were not selective to a single level but overarching in nature and were not perfectly equal across groups. Figure 1 provides a simplified visualization of the main findings, clustering the themes (shown in circles) by relevant stakeholder group (x-axis) and respective level (y-axis).

The following sections give a detailed overview about the separate themes. Participant identification numbers are used below to indicate direct quotations in accordance with the consolidated criteria for reporting qualitative research (COREQ) [48].

### Lack of knowledge about autism

The theme “lack of knowledge about autism” in adulthood was mentioned by all participants as an important barrier in healthcare of autistic adults at all three levels (individual, professional and structural; see Fig. 1). For this theme, several subthemes were identified in the data: (i) stigma about ASD in society, (ii) lack of knowledge among healthcare providers, (iii) research about ASD in adulthood and in women.

First, *(i) stigma about ASD in the society* and “that public and media often have a very distorted picture of what autism actually is, and especially the area of high-functioning^2^ is extremely difficult” (autistic adult; ibew6). According to participants, the portrayal of autism in the media has a negative impact on the healthcare of autistic adults because of misconceptions developed by stakeholders in the healthcare system.

Second, all stakeholder groups saw *(ii) a lack of expertise among healthcare providers* in adult autism as another major barrier. This “cluelessness” (autistic adult; ibew10) about autism would affect all professions in the healthcare system, e.g., physicians, psychotherapists, occupational therapists, and assistance providers. For example, autistic adults reported to hesitate sharing their diagnosis with healthcare providers because they feared discrimination. Participants considered the lack of education and training programs about autism to be the primary reason for this. It was “not easy at all” (healthcare provider; B1) to gain expertise in this field because healthcare providers “do not come in contact with any autism spectrum disorder treatment or even diagnostic theoretical idea, such as with anxiety disorder, personality disorder, etc.” (healthcare provider; B1). A psychotherapist described:

From my experience in advanced training in behavioral therapy, I can say that I certainly did not gain my knowledge about autism from advanced training in psychotherapy, so after six hundred hours of theory or something, I have already stopped counting and I have not had a single autism seminar, not a single one. […] I haven’t had a single teaching unit in my whole education on this, which is from my current perspective, absolutely devastating […]. (B4)

Participants described that healthcare providers frequently refused treatment based on lack of knowledge. For example, responses included “oh, you are autistic, no, I don’t have any experience with that, better contact someone else” (autistic adult; ibew11). Another autistic adult reported that “with Asperger’s and Attention deficit hyperactivity disorder [ADHD] in particular, I found that I had to make a lot of phone calls to find a therapist. I think nineteen out of twenty said, ‘we have no idea about that’” (ibew15). Autistic adults reported experiences like this and the subsequent burden. In addition, knowledge gaps were suggested as a reason for the high rate of misdiagnosis and resulting mistreatment of autistic adults. This was a prominent theme across all three stakeholder groups, but autistic adults and family/partners in particular reported stories of misdiagnosis, years of mistreatment, and resulting negative impact on a personal and emotional level. A man described his autistic wife’s diagnostic path:

[…] my wife was also diagnosed with all kinds of things in life: schizophrenia, depression, and so on. So almost every diagnosis, but no one had the idea that it could be autism and that’s just a pity that someone wastes her youth and her childhood. (Relative; I1)

Third, all three groups emphasized the need of further *(iii) research on ASD in adulthood and in women*. Participants demanded better screening and diagnostic tools for adults in general and especially for women, as it would allow sooner diagnoses and “filtering of waiting lists” (healthcare provider; B2). Furthermore, “girls *fall through the cracks*” (relative, I5) in mental healthcare because characterization and manifestations of symptoms often differ in women and they tend to be more adapted than male autistic persons:

[…] I think this is very regrettable. And I know many young women who were diagnosed in adulthood. Before that, they had been diagnosed with borderline or something else. I think that’s very bad for the young women. And I would like to see that, in general, neurodiversity […] is taken into account when it comes to burnout and depression.” (relative, I5).

In addition, healthcare providers identified the lack of valid measurements for severity assessment to be another field to be researched: “severity is poorly operationalized and therefore it is not possible to say in a very general way what the individual person needs in terms of support” (healthcare provider, B2).

### A need for increased participation/ involvement

The theme “a need for increased participation/ involvement” refers to both the autistic adults and the relatives which should become more involved and participate in different areas of the healthcare system, according to most participants. This theme is classified at both the individual and professional level (see Fig. 1).

On the one hand, autistic adults and relatives postulated to become better involved in examinations and treatment in general. They demanded to be directly involved in decisions and to be informed about fundamental issues, such as medication, diagnoses and interventions and recommended treatment to be as transparent as possible.

Moreover, autistic adults recommended to systematically involve autistic adults into knowledge dissemination and research on ASD:

What is very important to me is bringing education about autism into the broad field. […] This is about medicine, care, and that must be brought into the broad area, and definitely in participation with autistic people. Only from the view inside it can be explained what autism is. (Autistic adult; ibew11)

In addition, all groups highlighted that “it is important trying to have a stronger exchange with family members” (autistic adult; ibew8). Support of relatives and partners, e.g., in planning and/ or accompanying appointments or activities of daily living was essential according to the participants. Without this support, many autistic adults would not be able to participate in the mental healthcare system. Nevertheless, they acknowledged barriers in involvement of relatives and demanded to “take a supporting person with you without being ridiculed” (autistic adult; ibew 2). Healthcare providers described that relatives also have a great impact on diagnostics and therapy, but are rarely involved at the moment:

And the family is not involved, it is not even implemented by the insurance company. There are always a few sessions with relatives, but the family is not really involved. And I also find that totally difficult when adults from the spectrum then end up in such an ordinary behavioral therapy. (Healthcare provider; T4)

### Consideration of autism-specific needs in treatment

This theme describes challenges in accessing or participating healthcare services due to specific characteristics of autism itself. It addresses all relevant levels and was mentioned by all of the three stakeholder groups but was predominantly represented in the group of autistic adults. Following subthemes were identified in the data: (i) difficulties in executive functions and self-management, (ii) need for consistency and transparency, (iii) autism-specific sensory sensitivities, (iv) difficulties in verbal and nonverbal communication.

Participants reported *(i) difficulties in executive functions and self-management* on healthcare seeking behaviors affecting access to healthcare services, for example, when “completing very simple paperwork” (healthcare provider; B4), adhering to medication, navigating in the healthcare system, or planning/ attending appointments:

So, something like making an appointment, but also attending the appointment if I don’t know the place yet. That means estimating how long it will take me to get there, even with public transportation, because I don’t drive a car. So to organize something like that. Or, in general, not to take on too much for one day and to estimate how long I have to plan for this appointment. (Autistic adult; ibew2)

Furthermore, the *(ii) need for consistency and transparency* in treatment and healthcare settings was emphasized. “New situations are difficult” (autistic adult; ibew 14), including changes in staff, appointments or environment. An autistic adult described:

The biggest challenge is when I go to a new physician where I don’t know the environment, the practice, everything. To get used to it is a big challenge for me, for which there is not really a solution apart from doing it. (Autistic adult, ibew5)

Autistic adults said that it would be difficult not to be involved in treatment and not to obtain “background information”, for example about physical examinations (autistic adult; ibew11). Some said it was helpful if providers “explained [treatment] step by step”, if they involved in medication adjustments, and informed about diagnoses.

Most participants highlighted the impact of *(iii) autism-specific sensory sensitivities* on seeking or receiving mental healthcare for autistic adults. Healthcare facilities would cause stress and overload because they are not tailored to autistic peoples’ needs. For example, it was described that “physical contact is unpleasant” (autistic adult; ibew8), “smells can be very disturbing and irritating” (autistic adult; ibew4), “noises are very unpleasant and hardly bearable” (autistic adult; ibew2), “sensitivity to light leads to distraction” (autistic adult; ibew4), and overall “chaos reigns in the practices” (autistic adult; ibew 14). In addition, difficulties in body awareness and pain perception were mentioned:

On the one hand, being very sensitive during examinations and being unable to handle and endure a lot of situations, but on the other hand, not always noticing when there is pain or symptoms. In any case, these are also major barriers. (Autistic adult; ibew2)

Autism-related *(iv) difficulties in verbal and nonverbal communication* with medical staff also were a prominent topic, such as difficulties in making appointments by phone or miscommunications with healthcare providers during treatment or examinations. Autistic adults reported to struggle in following spoken instructions or answering open-ended questions. For example, an autistic participant described the following interaction with a physician:

“‘Where exactly does it hurt?’” - I always feel very stupid when I can’t answer that. It would be better to ask, “Is this a pain in the whole abdomen or specifically in one spot?“ Then it would be easier for me to understand that it may just hurt in the whole abdomen and that this is okay as a description. (Autistic adult; ibew12)

In addition, autistic participants described problems in communicating symptoms because “they like to be on the matter level and tend to communicate information and the emotional stuff doesn’t get carried along” (autistic adult; ibew3). This would cause that “many symptoms are not even recognized properly or are dismissed as incidental […]” (autistic adult; ibew4). Autistic adults also described difficulties with typically very brief clinical appointments: “I feel rushed under time pressure and then I forget a lot of what I actually wanted to discuss. The appointments are often too short” (autistic adult, ibew9).

As mentioned before, this theme was predominately represented in the group of autistic adults. In general, they emphasized individualization of treatment of autistic adults as a facilitating factor to cross barriers in these autism-related difficulties.

### Lack of services

All stakeholder groups commonly agreed upon a lack of mental healthcare services for adults with autism: “The whole system is not intended for autistic adults. It seems as autism is seen as a children’s disease […]” (autistic adult; ibew6). Especially “the high-functioning are not acknowledged or are not seen as a relevant group” (autistic adult; ibew6). Following subthemes were identified, referring to the professional and structural level: (i) transition, (ii) diagnostic services, (iii) psychotherapeutic services, (iv) inpatient services, (v) low-threshold services, (vi) assistance services.

Participants criticized the lack of adequate support systems for *(i) transition* from youth into adulthood. Mental healthcare would be provided during childhood and adolescence, but collapses once autistic individuals reach adulthood. An autistic adult reported:

The main problem is that the areas are separated from each other. The area of child and adolescent psychiatry and the area of adult psychiatry, not only in psychiatry, but also in medical care. Then you have to go from one to the other and are and treated completely differently. (Autistic adult, ibew2)

Participants demanded transitioning being adjusted in regard to the development of the individual rather than to the age and to “install a support system at least temporarily” (healthcare provider; B4).

Another prominent topic, particularly discussed by healthcare providers, was limited *(ii) diagnostic services* for adults seeking an ASD diagnosis as expressed by long waiting times for obtaining diagnostic assessment:

In [city name], healthcare is, I would say horrible, waiting times at the [name of hospital] for the autism consultation two and a half years, only a handful psychiatrists in practice, completely overloaded and waiting times about three years. (Healthcare provider; B2)

This was explained by a limited amount and limited capacity of specialized services: “there are not enough diagnostic and therapeutic centers at all” (healthcare provider; T5), especially in rural areas. Furthermore, it was expressed that psychometrically valid and specific screening and diagnostic instruments for autistic adults, also suitable for use in primary care, need to be developed which could shorten or optimize the diagnostic process.

After ASD diagnosis, participants reported “being left completely alone” (autistic adult; ibew13) with insufficient availability of subsequent *(iii) psychotherapeutic services*. Participants also described limited and scattered specialized services (“in the country side, very very few therapy options for older autistics or Asperger’s autistics […]. I would have to drive 100 kilometers to get anything, any help at all.“ (relative; I4)). Autistic adults and relatives remarked to put up with long-distance therapy because there were no services close to their residence or waiting lists were at maximum capacity. Reported waiting times for psychotherapy ranged from nine months to one and a half years. Even when successfully accessed therapy, a few autistic adults highlighted their need for individually tailored support and personalized treatment: “The needs are very individual. And even in the high-functioning area, if you compare two people, then the needs or the level of suffering, etc. and the living situation might be very different (healthcare provider; B3).”

With respect to *(iv) inpatient services*, including psychiatric or rehabilitation services, all stakeholder groups agreed upon the need for increased specialized and personalized services. “In inpatient healthcare of people with autism spectrum disorders, […] there are very, very few offers” (healthcare provider; B2), and “shared rooms in hospital are just not acceptable. I think if you are autistic, you should have the opportunity, at least if it is possible, to get a single room” (relative; I2).

Furthermore, all groups of stakeholders criticized a lack of *(v) low-threshold services*, suggesting, for example, to expand self-help groups for autistic adults as well as for relatives:

Very little attention is paid to the fact that not only the affected person is impacted, but also a large number of people around them. I think it would be very, very good and very important if more counselling were created, for example get relatives informed or they can also experience support for themselves. (Autistic adult; ibew15)

Relatives/ partners described stress and burden due to the support of the autistic relative. Therefore, they “would really like to have support also as a relative. That you are taken by the hand in order to understand conflicts better, to accept peculiarities better and to spend a good and stress-free life together with the affected person” (relative; I2).

Autistic adults highlighted the need for further low-threshold services, such as music, sports, or occupational therapy, specialized for autistic adults or low-threshold crisis counselling: “there is only one telephone emergency service for autistic people in Germany and that is a voluntary one. And that’s also relatively small given such a high suicide prevalence” (healthcare provider; T3). Counselling services for different purposes were mentioned. Healthcare providers described the need especially for social therapeutic services in particular: “because often it’s not about psychotherapy or processing the experiences from the past, but supporting, in order to somehow cope with the daily challenges, for Asperger’s, […] with the social challenges” (healthcare provider; B2). The need for peer counseling and information about existing services was also emphasized. Lastly, the lack of services for those who are not able to get manifest therapy or are waiting for it was expressed:

I think a larger network of counselling centers would be helpful, because I think that many affected people, many relatives experience greater barriers finding information at a clinic, a therapy center, than going to a counselling center or to a regulars’ table and seeking advice and exchange there. So, I think that would be a practical way to facilitate initial contact. (Autistic adult; ibew5)

Furthermore, participants across all groups highlighted the lack of *(vi) assistance services* for autistic adults without ID “because autism doesn’t mean therapy will be done and then everything will be okay again” (relative; T6). It was emphasized that case management (i.e. services to provide adequate healthcare for autistic adults according to their individual needs) needs to be implemented to relieve the burden on autistic adults, but also on relatives who often provide care and assistance. An autistic adult responded to the question how relatives and partners could be supported: “others who provide required assistance. In other words, assistance for autistic people across the whole lifespan. In my opinion, even a high-functioning autistic person like me needs some kind of lifelong assistance service” (autistic adult; ibew 11).

### Limited access to services

Not only the lack of services was described, but also barriers in accessing the available services were highlighted in most interviews and focus groups. Two subthemes were identified in the data, which are loading on the professional and structural level: (i) lack of transparency about healthcare services, (ii) funding.

On the one hand, the *(i) lack of transparency about healthcare services* was described. Neither autistic adults nor relatives or healthcare providers were fully informed about available services. A healthcare provider described: “That’s the problem, caregivers don’t have an overview of available services, including myself. It’s not easy to find one’s way in this network, but that’s actually the most important thing” (healthcare provider; B1). For example, patients often do not receive adequate care after diagnosis and have to obtain information on their own or with the support of relatives. Those who found services reported to have problems with the complex application process that could not be managed without external support. Healthcare providers reported regularly assisting autistic people with application forms, even though they are not responsible to provide assistance services. Furthermore, autistic adults reported that, based on the division of healthcare services among different social systems, navigating the healthcare system would be difficult:

Access to services is so massively divided among the various social systems by law that it is incredibly difficult for me to even find out who is responsible for me and from whom I have to apply for what. Yes, the fact that the individual social systems are always trying to pass the buck to each other is also not conducive. (Autistic adult; ibew4)

In relation to this, it was criticized that *(ii) funding* of autism therapy is not provided by health insurance. An autistic adult shared her opinion on this topic:

Because at the moment autism therapy and psychotherapy are separated from each other. And a classic autism therapy is also not funded by the health insurance, because with health insurance financing, this healing aspect is always included, which is a wrong approach, in my opinion. (Autistic adult; ibew2)

Regular psychotherapy, on the other hand, is not funded by health insurance when only ASD is diagnosed. To be approved for psychotherapy, a mental disorder such as depression or anxiety disorder must be present. A healthcare provider mentioned “almost three-quarters have a relevant co-morbidity, so the largest group finds access” (healthcare provider; T5) to psychotherapeutic services, but it would be a problem anyway. In addition, it was criticized that statutory health insurance companies do not enable rapid support in case of crisis situations.

### Improvement of stakeholder collaboration

This theme includes the collaboration of relevant stakeholders in the healthcare of autistic adults on a professional and structural level, which were mentioned to be healthcare providers from various professions. Primarily healthcare providers, but also few autistic adults emphasized the need for collaboration between different groups of professions and “multi-professional teams work[ing] with affected people” (autistic adult, ibew7). Many participants described that this could allow for simplification of healthcare processes, as all providers can be on the same level of knowledge about the patient - as long as the autistic adult consents. Some participants mentioned that collaboration would also facilitate the transition between pediatric and adult healthcare providers:

That the new doctor can also call the old doctor if there are any questions until the autistic person is really back at home with the new one, where he or she is again in safe hands during this transition phase. (Autistic adult; ibew11)

Few healthcare providers also highlighted that exchange with other providers also contributes to increase knowledge about autism: “there should actually be more networking and more exchange of experiences” (healthcare provider; B2).

## Discussion

To our knowledge, this is the first comprehensive investigation including the perspectives of autistic adults, relatives and healthcare providers on barriers and needs at the individual, professional and structural levels of mental healthcare for autistic adults without ID. We identified six major barriers to mental healthcare for autistic adults in Germany, which primarily affect autistic adults but are also of concern for relatives and healthcare providers: (i) lack of knowledge about autism, (ii) a need for increased participation/involvement, (iii) consideration of autism-specific needs in treatment, (iv) lack of services, (v) limited access to services, and (vi) improvement of stakeholder collaboration. The majority of the themes are in line with existing literature, focusing on general healthcare in the U.S [26]., thus, previously reported barriers similarly apply to mental healthcare.

The results of this study partially replicated the three previously proposed levels of barriers (see Fig. 1). In addition to these three levels, we included the stakeholder group that is mainly affected by the existing barriers with the goal to give an overview about potential adjustments. Some themes were not presented in previous models, therefore we tried to integrate them based on existing literature and the qualitative study results. In line with previous research, “lack of knowledge about autism” and “consideration of autism-specific needs in treatment” were present on all levels [26]. These barriers have implications for all requested stakeholder groups. “Lack of services” and “limited access to services” were previously defined as barriers on the structural level. Based on our data, the professional level also seems to be important. Over the last years, participation and empowerment became more important in the area of mental healthcare including autism [37, 38] and was not considered as barrier in mental healthcare so far. Based on our data, the theme “a need for increased participation/involvement” was interpreted as the relation between autistic individuals including their relatives/partners (individual level) and healthcare providers (professional level) and has implications for all stakeholder groups. Likewise, “stakeholder collaboration” was not considered as specific barrier [26]. Previous research on interprofessional collaboration in the treatment of autistic individuals showed that cross-disciplinary collaboration can lead to improved patient care and maximal outcomes by capitalizing on the varying expertise, as professionals from a broad range of disciplines are needed to address the heterogeneous core symptoms and co-morbidities [49]. This has mainly implications for the healthcare providers themselves.

The identified barriers and stakeholder groups may apply for both national and international mental healthcare of autistic adults, since ubiquitous barriers seem to exist for adults with autism across countries and healthcare systems. This is supported by the fact that our findings are in line with previous evidence from various countries, e.g. United States [26, 30, 50], Canada [29], and United Kingdom [15, 51]. A common feature of all countries is the high level of healthcare expenditures by international standards, ranging from 17.7% of the GDP in die United States to 10% in the United Kingdom [32, 52, 53]. Thus, barriers to healthcare are stable across these countries, despite comparatively large amounts of funding being invested into healthcare. Therefore, the results of the current study could be of global appeal and point to specific areas for improvement, which could guide future development of needs- and evidence-based services and guidelines of mental healthcare for people with autism across the lifespan.

Policy-makers need to increase the availability of formal healthcare services and, in turn reduce healthcare inequalities by providing the necessary resources. For example, diagnostic and psychotherapeutic services need to be expanded in both urban and rural areas. Transitional periods (e.g., transitioning from adolescence to adulthood) need improved coordination. In general, healthcare facilities and processes should be more accessible for autistic adults, e.g. communication pathways. Most important, transfer of knowledge about autism needs to take place in the community to reduce stigma of autistic individuals, to train healthcare providers and inform about skills and behaviors necessary to provide respectful and adequate mental healthcare for autistic adults. In light of the massive burden autism represents for autistic individuals, relatives and society, research on ASD (e.g., symptomatology of autistic girls and women, screening and diagnostics of autistic adults, burden and support of relatives and partners) and its funding needs improvement. Education, research, and treatment should be addressed through the participation and collaboration of relevant stakeholders. In autistic individuals, symptom expression and symptom severity vary both inter- and intrapersonally, so mental healthcare has to be flexible and tailored to the state of the condition. Findings indicate that individualized and personalized healthcare would provide best practice for autistic individuals [34, 54].

### Limitations and future directions

A major strength of the current study is the participatory approach, which ensured involvement of autistic peer researchers and relatives as well as service providers at all stages. Participation, which we had identified as important theme, in the current study was further maximized by offering several methodological participation modes to autistic adults (i.e., by e-mail or chat). The use of a purposeful, maximum variation sampling strategy allowed to obtain rich, comprehensive data from a wide range of participants. However, as with most qualitative studies, the sample does not represent a random selected sample of population and the sample size is limited. Moreover, it was not possible to recruit participants from some federal states (e.g. Brandenburg, Saarland; see Table S1). In addition, generalization of results across the entire autism spectrum is limited due to the lack of participants with ID, which may have reported different support needs. Nonetheless, their perspective was brought into focus groups by professionals, who also worked with autistic adults with higher levels of support needs. Furthermore, due to the COVID-19 pandemic, interviews and focus groups were conducted via video communication. Hence, it is possible that there might be a loss of subtle information that would have been presented in personal face-to-face settings. Lastly, stakeholder groups were interviewed using different settings (one-by-one interviews with autistic adults and focus groups with relatives and healthcare providers). Even though data collection and data analysis were carried out in a standardized manner independent of the setting, it cannot be entirely excluded that results were affected by this.

As manifestation of symptoms, severity, and secondary impairments vary widely between autistic individuals, further research should focus on the development of an individualized, needs- and evidence-based, severity-adjusted healthcare model. To allow an appropriate scaling of this services reliable epidemiological data from Germany will be needed [33]. To this end, within the scope of the *BarrierfreeASD* project, a follow-up large-scale online survey will be conducted to collect quantitative data in larger samples and provide a representative overview of the healthcare situation in Germany [36]. This mixed-methods approach aims to obtain a complete set of information to develop specific recommendations for a future healthcare model of autistic adults. Ultimate aim will be to develop recommendations for reforms and changes at all levels to improve mental healthcare of autistic adults in order to enhance independence and quality of life, for both the autistic adults and their relatives.

## Electronic supplementary material

Below is the link to the electronic supplementary material.

Supplementary Material 1 Table S1 Sample distribution by region and federal state

## Data Availability

The datasets used and analyzed in the current study are available from the corresponding author on reasonable request.

## Footnotes

1 We use the identity-first language, but equally support the person-first language. For further information, see https://autisticadvocacy.org/about-asan/identity-first-language/.

2 The authors state that this is a verbatim quote, although they take a critical view of the use of the term “high-functioning”. For further information, see https://pubmed.ncbi.nlm.nih.gov/31215791/.

## Abbreviations

ASD: Autism Spectrum Disorder
ADHD: Attention Deficit Hyperactivity Disorder
COREQ: Consolidated criteria for reporting qualitative research
ID: Intellectual Disability
GDP: Gross Domestic Product
N: Sample size
SD: Standard deviation
WHO: World Health Organization
