# Supplementary material for: Barriers and needs in mental healthcare of adults with autism spectrum disorder in Germany: a qualitative study in autistic adults, relatives, and healthcare providers
Source: BMC Psychiatry. 2023 Jul 21;23:528. doi: 10.1186/s12888-023-05026-x (PMC10362719; doi:10.1186/s12888-023-05026-x)
Supplement: Supplementary file 1 — Supplementary Material 1 Table S1 Sample distribution by region and federal state [file 12888_2023_5026_MOESM1_ESM.docx]

**Supplementary material**

| **Table S1** | | | | |
| --- | --- | --- | --- | --- |
| *Sample distribution by region and federal state* | | | | |
| Regions/ states | Autistic adults (n = 15)  *N* (%) | Relatives  (n = 12)  *N* (%) | Healthcare providers (n = 15)  *N* (%) | Total  (n = 42)  *N* (%) |
| **Northern Germany** | **7 (46.67)** | **7 (58.33)** | **6 (40)** | **20 (47.62)** |
| Bremen | 1 (6.67) | 0 | 0 | 1 (2.38) |
| Hamburg | 3 (20) | 6 (50) | 4 (26.67) | 13 (30.95) |
| Lower Saxony | 1 (6.67) | 0 | 1 (6.67) | 2 (4.76) |
| Schleswig Holstein | 2 (13.3) | 1 (8.33) | 1 (6.67) | 4 (9.52) |
| **Eastern Germany** | **1 (6.67)** | **0** | **6 (33.33)** | **7 (16.67)** |
| Berlin | 0 | 0 | 5 (26.67) | 5 (11.9) |
| Brandenburg | 0 | 0 | 0 | 0 |
| Mecklenburg-West Pomerania | 0 | 0 | 0 | 0 |
| Saxony | 1 (6.67) | 0 | 1 (6.67) | 2 (4.76) |
| Saxony-Anhalt | 0 | 0 | 0 | 0 |
| Thuringia | 0 | 0 | 0 | 0 |
| **Southern Germany** | **5 (33.33)** | **2 (16.67)** | **1 (6.67)** | **8 (19.05)** |
| Baden Wuerttemberg | 3 (20) | 0 | 0 | 3 (7.14) |
| Bavaria | 2 (13.33) | 2 (16.67) | 1 (6.67) | 5 (11.9) |
| **Western Germany** | **2 (13.33)** | **2 (16.67)** | **3 (20)** | **7 (16.67)** |
| Hesse | 1 (6.67) | 0 | 1 (6.67) | 2 (4.67) |
| Northrhine-Westphalia | 1 (6.67) | 2 (16.67) | 2 (13.33) | 5 (11.9) |
| Rhineland Palatinate | 0 | 0 | 0 | 0 |
| Saarland | 0 | 0 | 0 | 0 |
